# Feminism in the borderscape: Juarense women against injustice

**DOI:** 10.3389/fsoc.2024.1391529

**Published:** 2024-10-28

**Authors:** Asma Mehan, Natalia Dominguez

**Affiliations:** Huckabee College of Architecture, Texas Tech University, Lubbock, TX, United States

**Keywords:** activism, women's rights, social justice, Ciudad Juarez, machismo, gender violence

## Abstract

This article critically examines the feminist movement in Ciudad Juárez, Mexico, highlighting the struggles and activism of Juarense women against social injustices, particularly those exacerbated by machismo, the Narco War, and the manufacturing industry. The analysis explores the roots of machismo in Mexican culture, the impact of the maquiladora industry on women's lives, and the rise of feminist activism in response to these challenges. Emphasizing the intersection of gender violence and legal frameworks, the article incorporates feminist legal theory to argue for substantial legal reforms to combat the normalization of machismo and feminicide. It further discusses the resilience and determination of Juarense women, portraying their efforts as part of the broader global feminist movement.

## Introduction

Ciudad Juárez, a vibrant yet troubled border city in Chihuahua, Mexico, is marked by social injustices, including gender violence. Multiple social issues such as “Machismo” (EntreMundos, 2019), and “micromachismos” (EntreMundos, 2019), the constant mobilization of people in the manufacturing industry and the “Narco War” (Staff, 2010) have led to multiple catastrophes that have unfortunately marked the lives of thousands of women in the area. The Feminist Movement, being a result of the gender violence induced by the Narco War, the systematic and communal behaviors pushed by Machismo, and the highly influential manufacturing industry which women have been extremely affected by, has become stronger and stronger in this part of the world over the last few decades (De Reufels and Huhle, 2022).

The feminist movement in Juárez emerged in response to the gender violence induced by these issues, strengthening over the past decades. Thankfully for the people who suffer from these casualties—and unfortunately that they were affected by these social issues in the first place—borderland women (Herrera, 2021) are acting by rising to the authorities demanding them to make changes to the current faulty system. Women have increasingly mobilized to demand justice through protests, legal reform, and social media campaigns, drawing attention to the urgent need for systemic change (Alizadeh et al., 2024).

Women in the borderscape have demanded a response from the authorities through protests on at least every Women's International Day which takes place on March 8th since 2016 (Ebrary, 2023), law suggestions such as legalizing abortion on a national level (Gallegos, 2020) and the mobilization of the movement through social media (Ebrary, 2023) to tirelessly inform the population, bringing awareness of these extremely tragic situations in the city. This fight has been one of the most harsh, violent, and drastic movements within the city. This academic article delves into the events that triggered this upheaval and examines the efforts of Mexican and Latin American women to enact societal changes, with a particular emphasis on Juarense women.

## Methodology

The authors, as scholars in architectural humanities and urban anthropology, aim to shed light on the spatial, economic, and structural conditions contributing to gender violence in Ciudad Juárez. Leveraging their fluency in Spanish, Italian, and English, they combine feminist legal theory with their expertise in urban studies and anthropology to offer a unique perspective on how these interconnected forces shape the experiences of women in the city and contribute to the ongoing feminicides.

**Figure 1:**
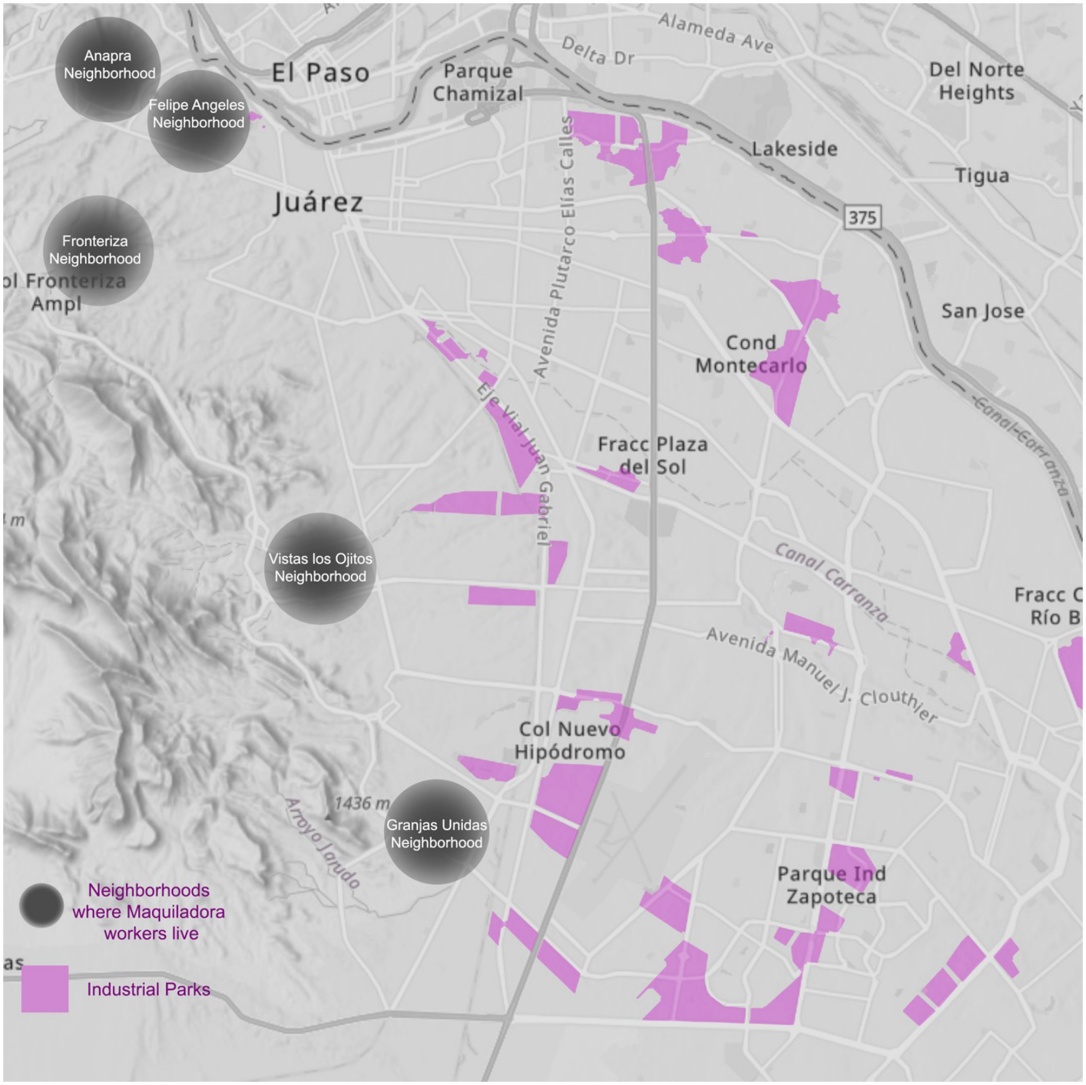
Map of maquiladora routes. Maquiladora workers live on the outskirts of Juarez, while the industries are found deep in the heart of the city. This means that the commute from home to work can be traitorous. Even with transportation provided by the industries, a lot of women find these routes dangerous, especially during the night. Multiple cases of missing women have been reported after last seeing them in their maquilas. Created by authors.

In this context, it is crucial to differentiate between the terms *femicide* and *feminicide*, both of which are used throughout this article. While often overlapping, these concepts carry distinct meanings that are critical to understanding the specific forms of gender violence in Ciudad Juárez and similar contexts (See Figure 1).

*Femicide* broadly refers to the killing of women because of their gender, a term that underscores the gendered nature of the violence but often lacks specific reference to the structural or institutional factors that may enable or perpetuate such acts. This term is frequently employed in international legal frameworks and human rights discourse, where it is understood as the extreme manifestation of misogyny and gender-based violence. It places the focus on individual acts of violence, often perpetrated by intimate partners or others within a domestic or social context. However, the use of *femicide* does not always consider the broader socio-political conditions that may allow such violence to persist, nor does it always address the systemic failures that can exacerbate these conditions.

**Figure 2:**
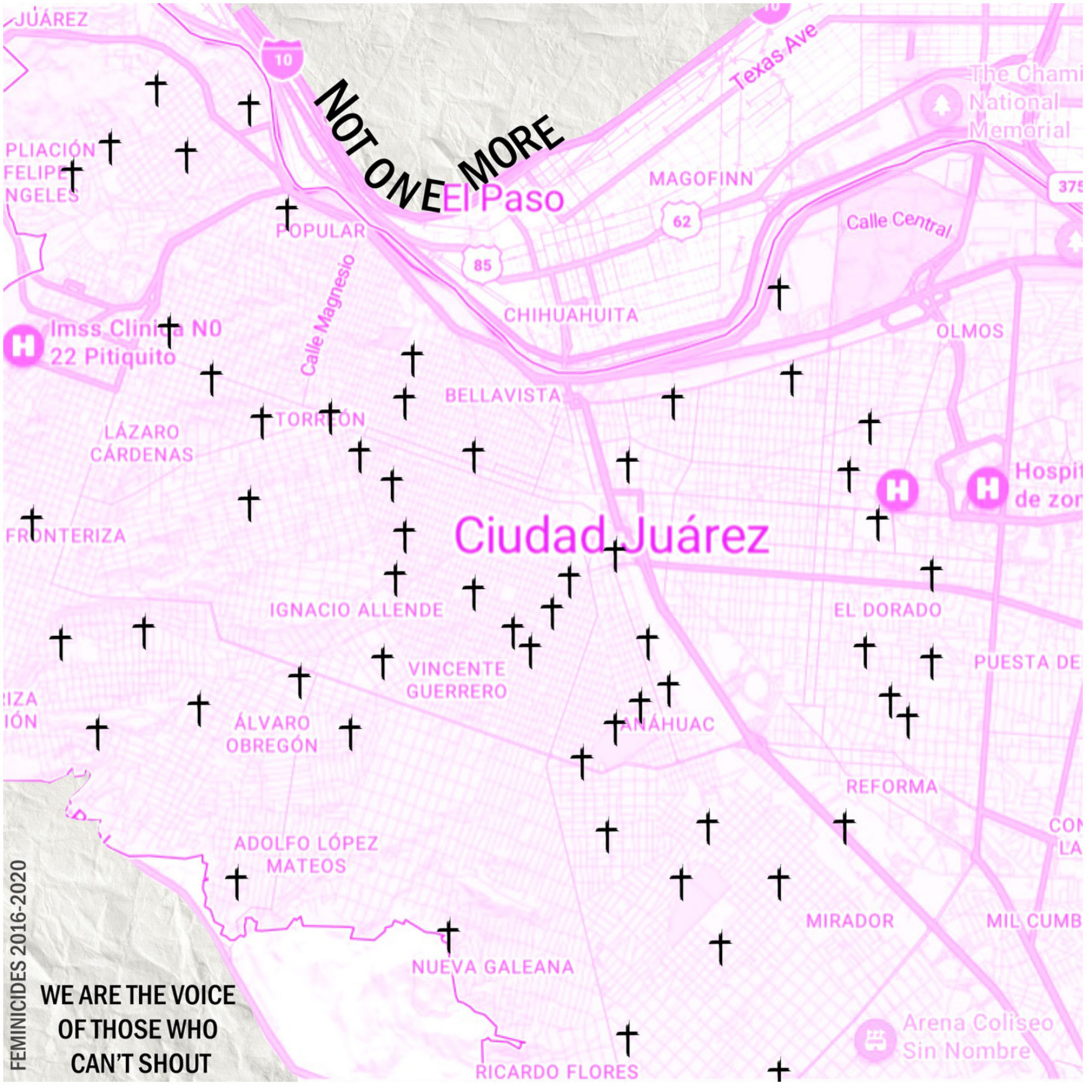
Cd. Juarez feminicide map. Ciudad Juarez has been known for its feminicides for the past two decades. During the years 2016 through 2020, locations where women were found dead were recorded by the reporter Maria Salguero. This map shows some of those locations. Most of them concentrated near the downtown area of the city. Created by authors.

On the other hand, *feminicide* is a more politically charged term, which goes beyond the individual act of violence to emphasize the complicity or failure of institutions, particularly the state, in preventing, prosecuting, and addressing violence against women. This concept, largely developed by feminist scholars and activists such as Marcela Lagarde, is especially relevant in contexts like Ciudad Juárez, where the persistence of gender violence is tied to a combination of patriarchal norms, impunity, corruption, and systemic neglect. By using the term *feminicide*, the authors aim to draw attention to the broader socio-political conditions—including legal frameworks, policing, and governmental responses—that have failed to adequately protect women and prevent such violence. *Feminicide* thus highlights not only the act of killing but also the structures that perpetuate impunity, neglect, and indifference (See Figure 2).

In Ciudad Juárez, the distinction between these two terms is especially significant, as the city has long been marked by both rampant gender-based violence and institutional failures to address it. The term *feminicide* is used intentionally to reflect the intersection of state failure, the normalization of machismo, and the economic exploitation of women in industries such as the maquiladoras, which together create an environment of pervasive insecurity for women. This approach allows for a more comprehensive critique of the conditions that enable gender violence in the city.

Their methodological approach integrates feminist legal theory with insights from architectural humanities and urban anthropology, using this interdisciplinary lens to investigate how the built environment, including urban planning and architectural design, impacts gender relations and violence (Varış Husar et al., 2023; Erek and Krasznahorkai, 2024). For example, the spatial organization of maquiladoras (manufacturing factories) and the broader urban landscape of Ciudad Juárez are critical to understanding the structural factors that contribute to gender-based violence and the barriers women face in accessing justice and safety. By analyzing how urban forms can either perpetuate or resist gender-based violence, the authors explore how spatial inequality, poor urban planning, and economic exploitation intersect with gender violence.

This methodology also emphasizes the role of physical spaces in shaping gendered experiences. For instance, the isolation of maquiladora workers, who often live on the outskirts of the city and must navigate long, unsafe commutes to their workplaces, is a key factor in the heightened vulnerability of women in Ciudad Juárez. These spatial dynamics, coupled with a legal system that has historically failed to protect women, create an environment where feminicide thrives. The feminist movement in Juárez, therefore, is not only a response to direct acts of violence but also a reaction to the broader socio-spatial and legal conditions that perpetuate gender inequality.

While previous studies have explored feminicide from various angles, this article emphasizes the spatial and structural dimensions of these injustices. By situating this discussion within both activist and academic contexts, the authors highlight the complex interplay between built environments, gender dynamics, and legal structures. This interdisciplinary approach aims to provide new insights that resonate with both activist and scholarly audiences, offering a more nuanced understanding of how gender violence is embedded in both physical and institutional spaces.

## Struggles and progress: women's rights movement in Latin America

To understand the feminist struggle in Ciudad Juárez, it is essential to recognize the broader historical context of women's rights movements in Latin America. From the late 19th to the mid-20th century, a constant mobilization of women throughout the region sought to push for social issues to be addressed by local and national governments such as labor laws, civil status, education, and political rights (Mehan, 2024c). These movements were diverse and encompassed a range of political ideologies and social classes (Mehan, 2024d).

It is important to note how the diversity of women's backgrounds and social issues they wanted to address became an important factor in the whole Latin American movement. Cora Fernandez Anderson in his academic article mentions how the movement encompassed multiple political spaces, from liberal to socialist movements, and how it involved women from different social classes (La Verdad, 2019). The creativity of women through all their struggles is shown by noting their various mediums to express their anger and discontent (Mehan, 2024e,f). During this time, multiple academic writings emerged from the discontent of governmental indifference toward these social issues (Nawratek and Mehan, 2020). The unconformity manifested in poetic and prose works and publications in women's magazines came from educated and elite women, while the middle class used the form of protests to manifest their increasing concerns about their educational opportunities and civil status (Rodríguez, 2014).

While the women's movement in Latin America emerged during the mid-20th century, labor movements coincided in the region. Thus, women from different professions, including domestic workers, launderers, and textile workers united with their male colleagues to demand maternity leave, fair wages, and better and improved working conditions. While this happened, women's suffrage was still not addressed until 1961 by every Latin American country. Through the years, we have seen a notable fight by all Latin American women seeking their rights to be implemented into the national governmental system, labor conditions and safety for women are still a very constant controversy that women in Latin America, specifically Juarense women have struggled with.

## Unveiling the layers of machismo: impact on women in Mexican society

Machismo, characterized by hypermasculinity and aggression toward women, remains deeply embedded in Mexican society and is a significant barrier to gender equality. “Machismo” comes from the word “macho”, meaning a man with exaggerated masculinity, oftentimes leading to aggressive and dominant behavior toward women (openDemocracy, 2012). This cultural construct has historically entrenched toxic masculinity, where dominance, control, and violence against women are normalized (Reuter's, 2010; Vallentin, 2009).

**Figure 3:**
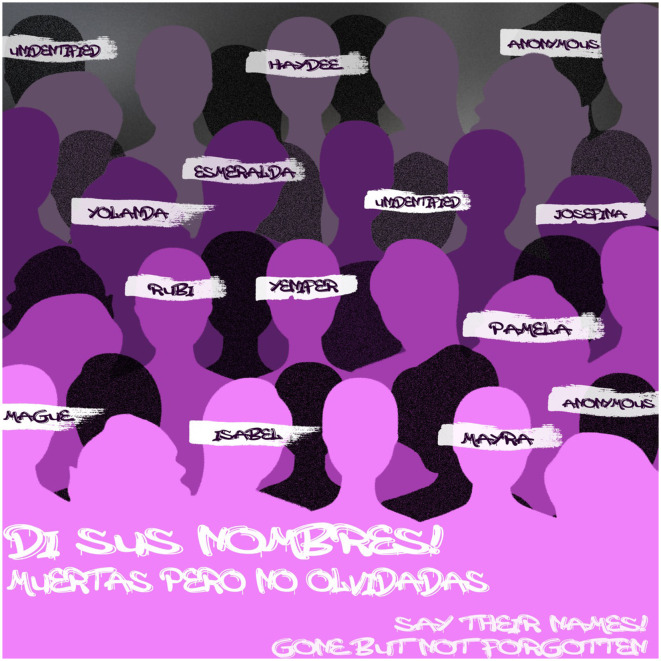
Victims of femicides. Feminists manifest during tough times, to demand the justice for the victims of feminicide. During feminist marches, the names and faces were plastered in the walls of the Municipal Institute of the women in the march for the International Women's Day in 2019 (8th of March). The poster mentions a few of the names of these women that were killed in Ciudad Juarez in the past three decades. Created by author. Inspired from: Paley (2020).

The widespread issue of toxic masculinity manifests in both micro and macro levels, from everyday interactions to systemic oppression. This ranges from the trivialization of women's emotions (e.g., calling them “crazy” or “too sensitive”) to more violent forms, such as feminicide, where women are killed because of their gender (De La Morena, 2020). These patterns reflect not only individual behaviors but a cultural norm that perpetuates gender inequality (Connell, 1987). This toxic masculinity, reinforced by societal expectations of male dominance, is also reflected in workplace environments, where women are often treated as second-class citizens (Lagarde, 2010). In Ciudad Juárez, this is particularly evident in the maquiladora industry, where women workers face both physical and psychological oppression, contributing to an environment that fosters violence against them (See Figure 3) (Monárrez Fragoso, 2002).

Feminist legal theory, as outlined by Simone (2024), provides a crucial framework for addressing these deep-rooted issues. Legal feminism emphasizes the importance of creating legal tools that challenge and dismantle sexist norms and practices that perpetuate violence against women. Incorporating this perspective, the article advocates for a more comprehensive approach to combating gender violence, recognizing the normalization of “machismo” as both a legal and social issue.

To fully understand how this affects gender inequality and gender-based violence, we must consider the concept of hegemonic masculinity, as theorized by Connell (1987). Hegemonic masculinity refers to the societal standards that position men as dominant, further reinforcing gender stereotypes and toxic masculinist behaviors. In the context of Ciudad Juárez, these behaviors permeate not only domestic spaces but public and institutional spheres as well, contributing to the systemic violence against women (Monárrez Fragoso, 2002).

Local feminist scholars such as Lagarde (2010) and Monárrez Fragoso (2002) have critically analyzed how patriarchal structures perpetuate violence, especially through the normalization of machismo and its institutional manifestations. Lagarde's theory of “captive women” explores how societal structures keep women socially and economically dependent, making them more susceptible to violence. Monárrez Fragoso's analysis of feminicide emphasizes the systemic failures of the state to protect women, where machismo shapes both gender relations and the broader culture of violence.

Not only do women have to fight against political incompetence from their government, but there is also a systematic fault that creates aggressive and violent behaviors in civilians. This has been a historical issue for centuries, starting around the colonization of Mexico, as previously discussed, and yet it is still a huge part of the interaction between genders not only in Juarez but at the national level too. Machismo on a small scale can look as simple as calling your girlfriend “crazy” or “too sensitive” when expressing her feelings, to something more violent like the killing of your girlfriend for speaking to other men. This type of behavior has become more and more usual to the point where the authorities recognize the murder of women, simply because they're women, as “Feminicide” (De La Morena, 2020). This term finally gave the seriousness of the effects of internalized and externalized machismo to the feminist fight.

**Figure 4:**
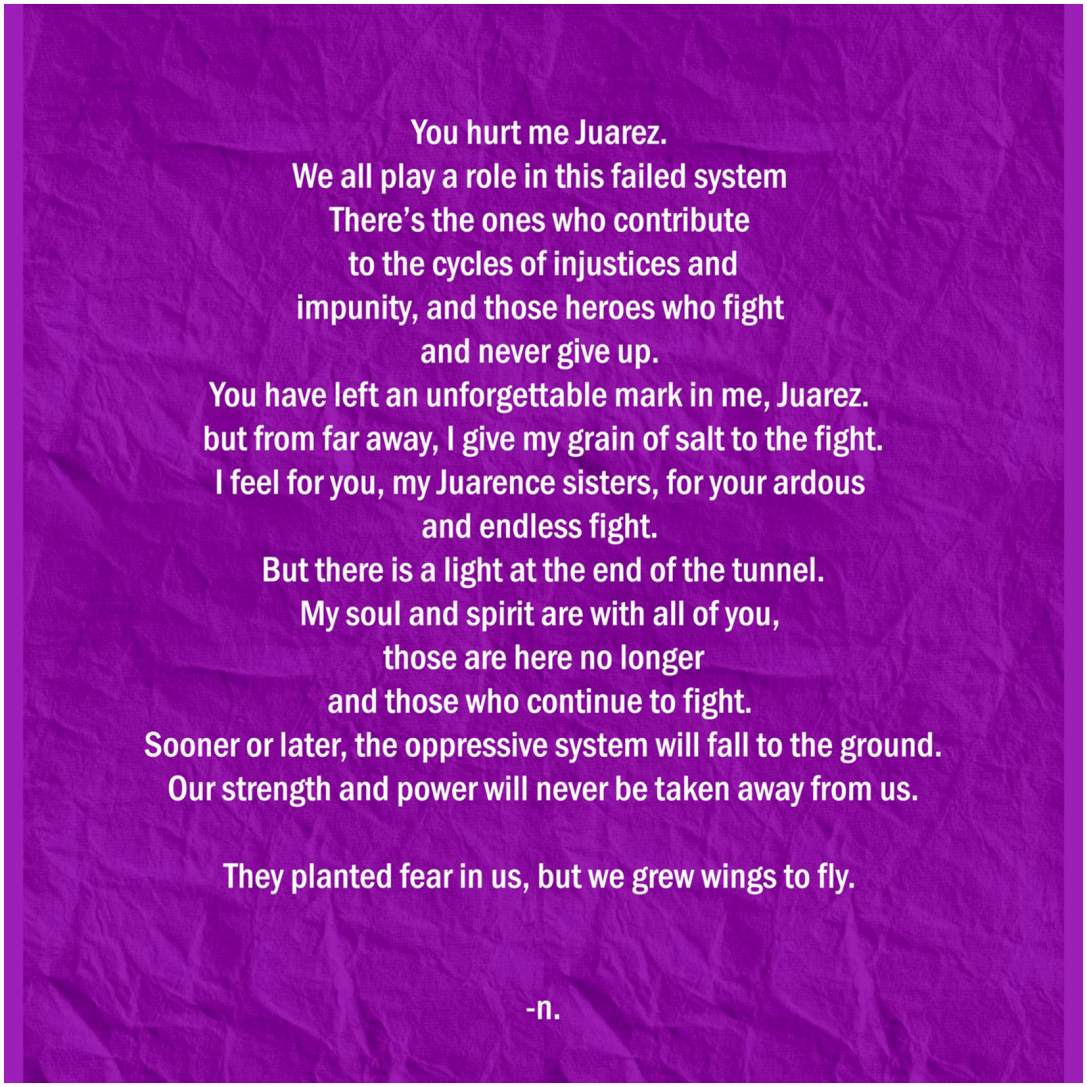
Poem for Juarez. The current situation in Ciudad Juarez has greatly affected its citizens. For those who have immigrated from the city, they find it hard to come back without the feeling of unsafety and uneasiness. This poem written by Authors came from a place of disturbance and anger when coming back to Cd Juarez. Nevertheless, the fight continues, and feminists of the area do not give up easily. Written and created by authors.

A violent act on a smaller scale is called micro machismo, which contributes to the overall gender violence happening in Ciudad Juarez. In a report on micromachismos (EntreMundos, 2019), four different aspects are identified as contributing to the widespread problem of gender violence in Mexico and across Latin America. The first aspect, “Utilitarian Micromachismo”, refers to the domestic environment and the traditional roles assigned to women within it, such as taking care of children and handling household chores. This reinforces gender stereotypes and restricts women's freedom and opportunities in both private and public spheres. “Hidden micromachismos”, refers to the imposition of the masculine “rights” of the men of the house, which oftentimes leaves women without a voice, rendering them invisible in the decision-making process in the household or a community (EntreMundos, 2019). This technique uses manipulation on women to keep following the path the men would want them to. Crisis-related micromachismos are used with the conscious unequal positions that women and men have in a relationship. For example, pointing fingers and assigning the fault of arguments on the woman, simply because men know they have the “upper hand” in the relationship. Multiple feelings like guilt, shame, and manipulation toward women are used with this micromachismo (See Figure 4). Coercive micromachismos (EntreMundos, 2019) refers to the use of moral, emotional, and economic power over women. The main impact that women have through this micromachismo, is the constraint of freedom to make decisions, such as forcibly wanting to pay for the bill on a night out or the imposition of “lady-like” attitudes that align in a patriarchal society. Phrases that are heard every day not only in Ciudad Juarez but in the whole country of Mexico, that could be taken as “culturally acceptable” contribute to the gender violence that women suffer. Sentences like “You fight like a girl,” “I can't stand her today, it must be that time of the month,” “she only got promoted because she slept with someone,” “dressed in those clothes, she was practically begging for it” add to this erroneous idea that men all over in Mexico have. Women in this part of the world, struggle with these societal-engrained issues every day of their lives, unfortunately, it is not only in Mexico, but the whole region of Latin America has had its fights and struggles as well.

## Maquiladoras in Ciudad Juarez: economic backbone and employment hub

The maquiladora industry, a key economic driver in Ciudad Juárez, involves manufacturing factories that import raw materials, process them, and export finished products to other parts of the world (Grajeda, 2023). Women, who comprise nearly half of the labor force, face numerous challenges, including workplace discrimination, unsafe conditions, and the constant threat of gender-based violence (Mexico Industry, 2021). It is crucial to consider how the spatial organization of maquiladoras and the broader urban landscape impact gender relations. For instance, the remote locations of many maquiladoras, often far from residential areas, force women to navigate unsafe commuting routes. These spatial conditions exacerbate the risks of gender violence and limit women's access to justice and support services.

Due to its proximity to the United States—the city of El Paso, TX in particular—creates an interconnected area, or “sister cities”, with well-established industrial and business clusters (Grajeda, 2023). Almost 60% of the economic contribution comes from maquiladoras in Ciudad Juarez (Grajeda, 2023), making it one of the biggest businesses in the borderland area. Maquilas in the area are primarily international companies, making Ciudad Juarez the lead provider of employment in this industry in the entirety of the country, providing more than 300,000 jobs and employing a significant portion of the city's workforce (Grajeda, 2023). Because of this, a constant mobilization of people from all over the Mexican country seek job opportunities in the region of Juarez.

Despite contributing significantly to the city's economy, female workers experience inequities that reflect broader societal norms rooted in machismo. There is a critical need for policy reforms that protect women's rights and ensure safe working environments, drawing on feminist legal frameworks to argue for substantial changes in labor laws and workplace practices (Maquiladoras, 2003).

## Maquiladoras in Ciudad Juarez: women's labor, challenges, and the feminist fight

Juarez has always been a city of immigrants, and one of the greater factors that pushed for immigration during the 1980s was the booming of the maquiladora industry (Ramírez, 2016). This industry offers a lot of incentives to push for immigration to the city such as free transportation from home and to work, meals provided during shifts and other seasonal bonuses (Ramírez, 2016). In an article by Israel Molina, a reporter for Mexico Industry Magazine, many people from different backgrounds, including young women and single mothers, who make up almost 36%, or almost a million women, of the maquiladora workers (Mexico Industry, 2021), have migrated from everywhere in the country to Juarez. Most of these industries are located deep in the center of the city or very close to its edges, while most of the workers live on the outskirts of the city, where water, electricity, and garbage disposal are very hard to reach (Maquiladoras, 2003), due to its remoteness compared to their employment location. A big concentration of maquiladora workers lives on the west side of the city, close to the mountains (Maquiladoras, 2003). According to a reporter of La Jornada Magazine, Rosa Isela Perez Torres, the disappearances and killings of women workers in the industry, are not taken a closer look by the international consortiums and they “feel they have no responsibility for the problem” (Maquiladoras, 2003). But seeing how the female workers for the maquiladora industry form almost half of its population, it should and is their clear responsibility to address.

Due to the size of the city, where the maquilas are located, and where most laborers live, they must take the transportation units 2 h before their shifts. This means, that the routes of commuting can be dangerous for many of these women. There have been many cases in which women disappear on their way to work or from home. According to a report for Debate Magazine, in 2019, Anabel Montañez Lopez was last seen getting out of her job, in one of the maquiladoras in the city. Her body was tragically found 9 days later without a sign of vitality (Audelo, 2019). Just Like Anabel, many more have been lamentably taken from their families and friends and left alone to die. Because of this, the manufacturing industry, or maquilas, has played a significant role in femicides in Juarez. It is infuriating to see how Juarense women struggle through so many adversities, in their own homes, in public areas, and even in their own work environments. It is important to note the importance of Juarense women in the Mexican workforce and their contribution to the city's economic growth. A report from 2022 by the local newspaper El Diario shows that 4 in every 10 employments are occupied by women.

Even though their contribution to the economy in the city is growing exponentially, women in the borderland still see various issues in their workplaces. Making up almost 50% of the laborers, women in Ciudad Juarez still encounter various inequalities compared to the Juarense men in the area. In a report for Just Associates.org from April 28th, 2023, the Luxembourg Pink Women Collective of Ciudad Juarez (Colectiva de Mujeres Rosa Luxemburgo de Ciudad Juarez) is striving to bring, inform, improve, and start a dialogue about the health and safety of labor conditions that women in the area have (Lo Laboral, 2023). With the help of this collective and other women's organizations, borderland women have a bright future ahead, but while powerful, conscious, and strong women bring that to the table, there are still various issues that women encounter in the workplace. Many find that the inequality that they face in the workplace is a huge part of their overall health and safety as women.

The workplace can also be a very toxic environment for mothers and young women in the borderscape area. An interview with a former female maquiladora worker (who is 51 years old and worked for the maquiladora industry for over 10 years), conducted by Natalia Dominguez on September 16, 2023, informed us how the top-to-bottom interaction is between men and women in this industry. When talking to the interviewee, various subjects such as the difference in salary, toxic masculinity, and the expectations that each gender has in the workplace were covered.

“As women, we felt more compromised to not miss any days for personal reasons compared to our male peers. It was easier for men to get permission from their supervisor to take some hours off or swap time during the shift because of personal matters such as attending a school meeting, a doctor's appointment. As for women, all those reasons to miss part of their shifts would make us less reliable in the company's eyes, which also means that that made us good candidates to lose the job. Most of my female peers did not take that risk”, she said.

This proves how the difference in treatment from men to women in the workplace can affect women in an exponential way. Most women that work in the maquiladora industry, are providers for their whole family, just like the interviewee was. It is obviously not fair how women are treated in the workplace.

“In the supervisor level, I was the only woman, in a group of 12 people, the rest of the company's female workers worked in lower positions like operators, warehouse staff and clerical level positions”, she said, “Most of the time, I was given instructions and expectations of duties that took more than 12- hour shifts for all my team, but I was still expected to get it done”.

The women in the maquiladora industry have harsher standards in the work environment that affects them not only in the physical level, but in the mental level too. The interviewee continued to describe her experience in the maquila saying, “it was super tough for me and my family. I was not able to attend various family events such as school meetings, school presentations, doctor's appointments and even spending time with my kids… I had to juggle all the pressure at work while still being a provider for my family and a full-time mom. And as for many women in my workplace, ended up divorced because of this”.

In addition to the testimony of the senior female worker highlighted earlier, further voices from women in different positions across the maquiladora industry add depth to this analysis. For example, Maria, a 25-year-old factory worker, shared her experience: “I leave home at 4 a.m. to catch the company bus, and it's always a risk. Many of my colleagues have been harassed or worse during these commutes, but we have no other choice. The factory is the only source of income for my family” (Grajeda, 2023). Another worker, Ana, highlighted the disparities in wages and treatment between male and female workers: “Even though I've worked here for 10 years, I'm still earning less than some of the men who started recently. They get promoted faster, and they don't have to balance work and family the way we do.” These testimonies underscore the layered forms of oppression faced by women in the maquiladoras, where they are not only underpaid and overworked but also face daily threats to their safety (Grajeda, 2023).

## Unveiling femicides: gender-based violence in Juarez, Mexico

Feminicide, the deliberate killing of women due to their gender, represents the most extreme manifestation of gender-based violence in Juárez (De La Morena, 2020; Offiong, 2021). This form of violence has been politically legitimized, often underpinned by the normalization of machismo. Feminist legal theory underscores the necessity of aligning formal equality with substantial equality, ensuring that legal protections translate into real-world safety and security for women (Simone, 2024). According to the World Health Organization (WHO), a significant majority of femicides are committed by current or former partners and often involve a history of domestic abuse, threats, intimidation, or situations where women have less power or resources than their partners (Offiong, 2021). However, these statistics often conflate the murder of women and children, complicating efforts to understand the specific patterns and impacts of feminicide (McInnes, 2024). There is an urgent need for policy reforms and legal interventions that address the systemic roots of feminicide, as recommended by international Charters of Rights and Conventions (Staff, 2010; openDemocracy, 2012).

## Unveiling femicides: the impact of the Narco War on Juarense women

Gender Inequality and gender violence manifested itself throughout the region of Juarez in the early 2000s. In 2001, the escapade of the powerful drug lord Joaquin “Shorty” Guzman (alias El Chapo) from a Mexican prison started the hectic era when he tried to seize control of Mexico's drug trade (Staff, 2010). By the following year, the Mexican law enforcement captured the Tijuana cartel's leader as well as one of his brothers, this event weakened the cartel's influence in the Narco War. In the following years, multiple battles between the armed forces and the members of the cartel left multiple civilian and law enforcement casualties. The initiative that former Mexican President Felipe Calderon imposed the minute he arrived in office in 2006 (Staff, 2010) was a pretended resolution to the war on drugs in Mexico. The timeline of events that happened right on the first day of his term, December 1, highlights the tempestuous and violent nature of his presidential term. In his efforts, former President Felipe Calderon declared the war on drugs to the cartels stationed in Juarez.

Militarizing every corner of the city (Staff, 2010), Calderon wanted an end to the business that was created by these gangs in the city. With a very blurred line between good vs. evil, civilians, especially women, were the group most affected by this. Authorities made no further changes to the security measures around the city to protect this already vulnerable group of citizens. War is usually conducted and created by men, but women usually take the fall and Juarense women during President Felipe Calderon's term were not an exception (openDemocracy, 2012). During his term, Felipe Calderon did not address the issues women have been suffering from, and the root of the problem is a failed justice system of an endless cycle of violence and unpaid crimes (openDemocracy, 2012). Laura Carson, a reporter of openDemocracy (2012), conducted a study with multiple female participants from the border area affected by the Narco War, where they found disparities in the justice system. According to her study, only 2% of crimes committed in Mexico, are successfully processed and the criminals responsible are imprisoned. This leaves a very untrustworthy system for the region's population. “Why in Mexico?”, a participant in Carson's study said, “Because they can get away with it.” (openDemocracy, 2012). Mothers are also the ones to look for their sons after cartel stand-offs, with nowhere to look and no response from a war that they did not participate in or have a say in openDemocracy (2012). It is fair to say that at every step, Juarense women have suffered a lot throughout the last three decades.

At the beginning of the 1990s, the bodies of multiple women appeared in a communal grave (Gallegos, 2020) and this was believed to be the doing of a possible serial killer or an active satanic cult in the area. The bodies were found in horrible conditions, something that no human deserves. Even if these were the speculations of authorities back then, this is still a reality for women in the borderscape (Guillén, 2022). It has slowly become a fight known to the rest of the world, bringing awareness of this issue to the masses. According to reporter Itzel Ramirez (Gallegos, 2020), the Executive Secretary of the National System of Public Safety in Mexico, Juarez received first place for having the most femicides in all 100 municipalities in the northern Mexican state of Chihuahua. This integer is a nerve-wracking place to hold, in any place in the world. If we take a closer look at the names and faces of the victims, then we can humanize them, and not leave them to be forgotten, to be a part of a percentage. On the 15th of January 2022 (Gallegos, 2020), two women were killed, dismembered, and left on the side of the road on the Juarez/Chihuahua highway. Their names: Tania and Nohemi (Gallegos, 2020). It is important to note their names and lives to respect their humanity and their dignity. Just like Tania and Nohemi, there have been thousands of women violented on the side of roads, or in municipal barren fields, left for dead, and even some are not found most of the time.

## Voices of resilience: activism and remembrance in Ciudad Juarez

The feminist movement in Juárez is characterized by its resilience and determination (Mehan and Dominguez, 2024; Tappert et al., 2024). Activists like Marisela Escobedo Ortiz, who fought tirelessly for justice for her daughter, and Luis Castillo, who continues to search for his missing daughter, exemplify the relentless fight against gender violence and impunity (The Three Deaths, 2020; Infobae, 2022). These stories, along with countless others, serve as powerful reminders of the need for legal reforms that hold perpetrators accountable and protect women's rights (Mehan, 2024a,b). The integration of feminist legal theory argues for a more robust legal framework that supports these grassroots efforts and fosters a culture of justice and equality (Paley, 2020).

Marisela Escobedo Ortiz became the face of the movement during the early 2000s (The Three Deaths, 2020). Her daughter, Rubi Frayre, disappeared after meeting her perpetrator Sergio Rafael Barraza Bocanegra. She was later found atrociously dismembered in a barren field in the outskirts of the city (The Three Deaths, 2020). Marisela Escobedo then started her long and tragic fight for justice for her daughter, demanding the authorities find the criminal and make them pay for his doings. After receiving a dismissive and disrespectful response from the local police, she decided that if she wanted to see the criminal in jail (The Three Deaths, 2020), she had to conduct the investigation herself. The determination she had from the moment Rubi disappeared, until the moment of Marisela's last breath, is recognized and supported by the whole feminist community in Ciudad Juarez. She became the martyr in the fight against femicides during the early 2000s in the area, giving women hope, strength, and a fight to continue for the new generation of activists. The documentary “The Three Deaths of Marisela Escobedo”, narrates the fight she went through for her last year and a half of her life. The name itself lets us know how hard her fight was. The first “death” took place when Rubi was found tragically dead, the second, when she took the case to the court and the jury decided to leave Rubi's perpetrator free with impunity, and her third, was when she was killed while doing a peaceful protest outside of the Municipal Palace in Chihuahua city. When watching the documentary, as an audience member we can all feel her grief and anger as if we were there with her the whole process.

Fourteen years ago, Esmeralda Castillo was last seen in downtown Juarez (Infobae, 2022), in the early hours of the morning. Her parents have not stopped looking for her ever since. Luis Castillo, her father, has attended every single feminist march since 2009, so much so that his picture wearing Esmeralda's face has circulated all over national news and social media. Usually, during feminist marches, women are the ones leading the protest, but Luis Castillo is the only man who walks in front of the protest (Infobae, 2022), supporting and demanding answers from the authorities of the whereabouts of his daughter (Infobae, 2022). He has become a legend in the feminist movement in Ciudad Juarez because of his perseverance and determination over the years. He has approached the three levels of the Juarense/Mexican government for any news about Esmeralda, but for over a decade, he has received nothing but bribery from the authorities to keep him quiet (Infobae, 2022). Luis, of course, has denied this failed attempt at bribery. The authorities are worried that his voice is becoming too loud, reaching too many people about Esmeralda's case. But she is not the only one. A record shows in 2022 (Infobae, 2022), that 10 women in Mexico are killed every single day, and Juarez takes a big percentage of that amount (Infobae, 2022). But because of activists like Luis Castillo and Marisela Escobedo, and the rest of Esmeralda's family, we have a certain degree of hope to continue the battle to eradicate the danger of women's safety in Juarez and everywhere in the world.

The use of public spaces for activism—such as marches, protests, and public art installations—highlights the importance of spatial justice in feminist movements. The physical spaces of Ciudad Juárez become sites of both oppression and resistance, where women reclaim their right to safety and justice. The way that activists such as the Castillo family and Marisela Escobedo demonstrate their anger and determination, is using the city as their white canvas to paint. During multiple feminists' protests, activists painted Juarez in pink and purple; the official colors of the movement recognized worldwide. Monuments of multiple historic figures are spray painted and written on to demonstrate the discontent that the whole women population feels with this social issue. These monuments most of the time represent a shady past that does not align with the present or the future that activists want for everyone. In 2019 (Gallegos, 2020), one of these marches took place in downtown Juarez, because of police brutality against women happening in the city. The big mass walked through the center of the city and finished the route in the Municipal Institute for Women (Instituto Municipal de la Mujer). Names of girls and women were roll called to remember them and to demand justice for all their disappearances and for their deaths. Through the manifestations of feminists, the stories of the victims will not be forgotten, and will not be part of a percentage, but they become the fire that lights up the fight of the feminist movement.

In our modern era, there are remarkable social activists utilizing today's technology to amplify their voices. Jessica Fernandez, hailing from northern Mexico, stands out by hosting insightful interviews in podcast form, available on platforms like YouTube and Spotify. Her podcast serves as a platform for guests to delve into critical feminist topics, ranging from gender violence and mental health to economic challenges affecting women. With over 80 episodes under her belt, Jessica Fernandez engages with a diverse array of guests. From female entrepreneurs to scientists, social activists, mothers of victims, and even the victims themselves, her podcast provides a safe and open space for these individuals to share their knowledge and personal experiences. Through these conversations, she creates an avenue for exploration and empowerment, shedding light on various aspects of the feminist movement and offering a platform for marginalized voices to be heard and understood. In an interview conducted by Heraldo de Mexico, he helps tell Jessica Fernandez's story. “At 24 years old, Jessica Fernandez Garcia is a well-known activist on social media, in which she uploads videos addressing issues related to women such as feminism, empowerment, and self-love, aiming to raise awareness about these issues in her followers, who range in the age group of 14–35 years of age” (De México, 2020). Jessica utilizes her social media platform to create informative videos aimed at educating the public about these critical issues and the ongoing efforts by activists to eliminate them. Her goal is to raise awareness and provide insight into these significant challenges, shedding light on the work being done by activists striving to bring about positive change. Jessica extends her impact beyond creating educational content; she actively engages through conferences held across Mexico (Valdez, 2022). These gatherings specifically target high school and university students, serving as platforms to inform the younger generation about pressing social issues (Valdez, 2022[96mm] a sense of empowerment among them for a brighter and more equitable future.

## Progressive strides and empowerment: transformative movements in Ciudad Juarez

Feminist collectives in Ciudad Juárez have been at the forefront of the struggle against gender-based violence, yet their relationship with state authorities has been fraught with tension (Saigol, 2016). While these groups demand justice for the countless women who have fallen victim to feminicide, state responses have often been inadequate or outright dismissive (Paley, 2020). This dynamic is not unique to Mexico.

Drawing comparisons with Saigol's (2016) analysis of feminist movements in Pakistan, where state support fluctuated based on political regimes, we see that the changing nature of governance can significantly alter the state's engagement with feminist collectives. In Ciudad Juárez, local feminist movements have had to contend with both a culture of impunity and state indifference, like what feminist groups in Pakistan have experienced.

In the past decade, feminist collectives in Ciudad Juárez have pushed for legal reforms such as *Ley Olimpia*, which targets digital violence against women (The Yucatan Times, 2021). However, much of the legal framework remains insufficient to address the systemic issues that allow machismo and gender violence to persist. Feminist groups continue to face roadblocks, as the state's response remains largely superficial, addressing symptoms without tackling the root causes of violence (Paley, 2020).

“Ley Olimpia” law draws its name from Olimpia, a woman whose private content was maliciously shared without her consent by an ex-partner (The Yucatan Times, 2021). Implemented in 19 out of Mexico's 32 states, Ley Olimpia stands as a shield to protect individuals whose intimate content has been shared across social media platforms without their permission. It's a law designed to safeguard anyone, irrespective of gender or sexual orientation, from such violations (The Yucatan Times, 2021). What's striking about Ley Olimpia is that it's emblematic of the feminist movement's inclusive approach. It transcends mere gender boundaries, advocating not only for women but for anyone who faces any form of violence. This broader perspective underscores that the battle for women's rights in Mexico is far from over. Yet, it's these very initiatives that fuel optimism and promise for the forthcoming generations. They represent a crucial step toward building a society that respects individual privacy, autonomy, and dignity, fostering a more just and equitable environment for all. Just like Ley Olimpia, the feminists across Mexico have seen the fruits of their work today.

Reproductive rights have been one of the fights women in the region have encountered to be unsatisfied by their government. Through various demonstrations of discontent and law suggestions, Mexican women have the desire to have body ownership for themselves and for every woman in the region. In more recent years, due to the arduous fights of feminist activists, the government is acting in a direction that favors women and their body ownership. In a pivotal move reported on July 8th, 2021, through the online magazine Expansion Politica, Claudia Sheinbaum, the head of the Mexican government, led legislation advocating for reproductive rights (Navarrete, 2021). Under the banner of Voluntary Interruption of Pregnancy (Interrupcion Voluntaria del Embarazo), this legislation advocates for the right to terminate pregnancies within the first 12 weeks (Navarrete, 2021), marking a significant stride toward granting women across Mexico autonomy over their bodies. What's particularly noteworthy is the government's acknowledgment of the prevailing gender inequality and its link to pervasive gender-based violence in the country. Within the same article, the legislation is seen as a responsive measure, addressing gender violence such as rape and sexual abuse. It introduces provisions allowing pregnancies of up to 20 weeks (or 5 months) to be eligible for this procedure in cases of rape, eliminating the necessity for evidence or formal complaints to access this right (Navarrete, 2021). This multifaceted approach reflects an enormous evolution in Mexico's legislative landscape, recognizing and accommodating the diverse and often challenging circumstances women face throughout the country.

In an article of La Nueva Verdad Magazine, published on September 1st, 2020, the reporter Victoria Rossi speaks about the struggles of women in the border to find support from their government to safely of through an undesired pregnancy (La Nueva Mañana, 2020). Women in this part of the world, are obliged to either travel very far distances to find a clinic to get the abortion procedure done, or simply stay with crossed arms without any help from their officials. An interviewee (who decided to remain anonymous) provides the necessary medication for an early pregnancy to be interpreted for women in Juarez (La Verdad, 2021). She distributes Misoprostol, a medication used generally for internal ulcers but is also a great resource for a clandestine abortion during the early stages (La Verdad, 2021). CC, the interviewee, buys the medication through acquaintances and offers the help needed through her social media (La Verdad, 2021). It is incredible to note how women in need, help each other in difficult times, when support and aid is nowhere to be found in authorities or officials. Along the side of CC, various defense organizations, resources online and online pharmacies, are trying to bring these resources for those who cannot easily access them. Although it is a very complicated and arduous process to go through, the sense of community among Juarense women is stronger than ever. Changes are starting to be made in the governmental system in Juares. Last year, 2023, was filled with victories for the women not only in Ciudad Juarez but also in the whole country of Mexico. The fight for reproductive rights has been one of the grandest demands that Latin American women have sought for the last few decades, and it has finally been addressed by the national government as a human right for individuals who identify as capable of bringing life into the world. The demand started in Argentina in the early 2000s (Hatto, 2023) when feminists gathered and fought peacefully for the legalization of abortion and the protection of reproductive rights for all Latin American women. Through their efforts, Latin American women have come together to legalize a safe and healthy interrupted pregnancy. It is now a national law in Mexico, for abortion to be unpenalized as of the 6th of September of this year (Hatto, 2023). At last, Juarense women, and Mexican women in general, can finally take a victory in their constant fight, which shows that constant mobilization in the movement, perseverance, and most of all, hope, do bring fruits of their fight.

## Conclusion and further discussions

The feminist movement in Ciudad Juárez exemplifies the strength and resilience of women confronting systemic gender violence. This article integrates feminist legal theory to highlight the urgent need for comprehensive legal reforms to address machismo, feminicide, and broader forms of gender-based violence. The struggle of Juarense women is embedded within a larger global feminist movement advocating for justice, equality, and human rights. Their efforts, if supported by robust legal frameworks, have the potential to create a safer and more equitable future (Khasraghi and Mehan, 2023).

Ciudad Juárez has long been perceived as a site of danger, shaped by the violent Narco War of the early 2000s, the migratory influx driven by the maquiladora industry, and the persistence of patriarchal social constructs such as machismo and micromachismo. These factors contribute to the daily violence experienced by women. However, feminist activism is challenging these perceptions and working to reshape the region's socio-political landscape. Feminists in Ciudad Juárez have mobilized to demand systemic change through legislative advocacy, protests, and digital campaigns, contributing to significant reforms such as *Ley Olimpia*, which addresses digital violence. Activists such as Marisela Escobedo Ortiz, Luis Castillo, and Jessica Fernandez have emerged as prominent figures in this movement, offering hope and inspiring future generations. The struggle faced by women in Juárez is not isolated but is indicative of broader societal challenges that require urgent attention.

To effectively address the culture of gender-based violence in Ciudad Juárez, several key reforms are essential. First, the state must strengthen its legal frameworks to protect women from both physical and economic violence, particularly in industries such as the maquiladora sector, where women face unsafe working conditions and unequal pay. Beyond labor reform, the government must extend protections to encompass public and private spaces, addressing the vulnerabilities women face in transit, at home, and at work. Feminist collectives should play an active role in policy development, as their intimate knowledge of the lived experiences of women is crucial for shaping effective responses. Comparative lessons can be drawn from feminist movements in Argentina and Chile, where collective input has shaped national policies on reproductive rights and gender equality. Additionally, public campaigns must target toxic masculinity and entrenched gender stereotypes, with educational initiatives aimed at deconstructing machismo and promoting gender equality in schools and workplaces.

While significant strides have been made by the feminist movement in Ciudad Juárez, systemic challenges remain. The entrenched nature of gender violence, rooted in toxic masculinity, gender inequality, and state inaction, demands sustained advocacy and structural reforms. By incorporating feminist legal theory and centering the voices of local women, this article highlights the ongoing struggles while providing concrete policy recommendations to dismantle the culture of violence pervasive in the city.

This is a crucial moment to critically examine the social issues that have long affected Ciudad Juárez. Education and awareness are powerful tools in addressing the deep-seated history of gender violence that characterizes the region. By understanding the root causes and complex dynamics of this violence, activists and policymakers alike can generate meaningful and sustainable change. The activism of figures such as Jessica Fernandez, Luis Castillo, and Marisela Escobedo Ortiz demonstrates that collective action is key to exposing and confronting the realities of gender-based violence in Juárez. Progress requires a collective effort to recognize the widespread impact of these issues, and to channel this understanding into action that promotes systemic change.

The path forward involves more than awareness; it necessitates finding solutions and implementing innovative approaches to address these deeply entrenched problems. Cultivating a culture of respect, equality, and safety is essential for ensuring the security and wellbeing of all individuals in the community. Solidarity is crucial in this fight—by standing together and challenging the systems of violence and oppression, the community can create lasting change. A future in which every individual, regardless of gender, feels safe and empowered is possible, but it requires concerted effort. Ciudad Juárez and its border region can transform into spaces where security, equality, and opportunity thrive, free from the fear of violence or discrimination. The power to achieve this lies in the unity of collective voices and actions. Through continued advocacy, education, and solidarity, a just and equitable society can be realized for all.
